# Multi-parametric quantitative MRI reveals three different white matter subtypes

**DOI:** 10.1371/journal.pone.0196297

**Published:** 2018-06-15

**Authors:** Jack R. Foucher, Olivier Mainberger, Julien Lamy, Mathieu D. Santin, Alexandre Vignaud, Mathilde M. Roser, Paulo L. de Sousa

**Affiliations:** 1 Laboratoire des Sciences de l’Ingénieur, de l’Informatique et de l’Imagerie (ICube), CNRS UMR 7357, University of Strasbourg, Strasbourg, France; 2 Fédération de Médecine Translationnelle de Strasbourg (FMTS), University of Strasbourg, Strasbourg, France; 3 CEntre de neuroModulation Non Invasive de Strasbourg (CEMNIS), University Hospital, Strasbourg, France; 4 Department of Physiology, University of Strasbourg, Strasbourg, France; 5 CENIR, ICM, Paris, France; 6 CEA, I2BM, NeuroSpin, UNIRS, Gif-sur-Yvette, France; University of Virginia, UNITED STATES

## Abstract

**Introduction:**

Magnetic resonance imaging (MRI) shows slight spatial variations in brain white matter (WM). We used quantitative multi-parametric MRI to evaluate in what respect these inhomogeneities could correspond to WM subtypes with specific characteristics and spatial distribution.

**Materials and methods:**

Twenty-six controls (12 women, 38 ±9 Y) took part in a 60-min session on a 3T scanner measuring 7 parameters: R_1_ and R_2_, diffusion tensor imaging which allowed to measure Axial and Radial Diffusivity (AD, RD), magnetization transfer imaging which enabled to compute the Macromolecular Proton Fraction (MPF), and a susceptibility-weighted sequence which permitted to quantify R_2_* and magnetic susceptibility (χ_m_). Spatial independent component analysis was used to identify WM subtypes with specific combination of quantitative parameters values.

**Results:**

Three subtypes could be identified. t-WM (track) mostly mapped on well-formed projection and commissural tracts and came with high AD values (all p < 10^−18^). The two other subtypes were located in subcortical WM and overlapped with association fibers: f-WM (frontal) was mostly anterior in the frontal lobe whereas c-WM (central) was underneath the central cortex. f-WM and c-WM had higher MPF values, indicating a higher myelin content (all p < 1.7 10^−6^). This was compatible with their larger χ_m_ and R_2_, as iron is essentially stored in oligodendrocytes (all p < 0.01). Although R_1_ essentially showed the same, its higher value in t-WM relative to c-WM might be related to its higher cholesterol concentration.

**Conclusions:**

Thus, f- and c-WMs were less structured, but more myelinated and probably more metabolically active regarding to their iron content than WM related to fasciculi (t-WM). As known WM bundles passed though different WM subtypes, myelination might not be uniform along the axons but rather follow a spatially consistent regional variability. Future studies might examine the reproducibility of this decomposition and how development and pathology differently affect each subtype.

## Introduction

Although grossly homogeneous, brain white matter (WM) is known for its macro-organization in fasciculi and inhomogeneous signal in several magnetic resonance imaging (MRI) contrasts such as T_1_, T_2_*, and magnetization transfer (MT) weighted images [1,2]. Moreover, some WM diseases seem to have a tropism for specific regions, e.g. multiple sclerosis for peri-ventricular WM, adrenoleucodystrophy for the corpus callosum or lysosomal storage disease for posterior WM except for metachromatic leukodystrophy which affects anterior WM [3]. Lastly, several post mortem biochemical measurements have reported significantly different lipid content dependent on WM location [4–6]. Thus, there might well be different subtypes of WM.

Using multi-parametric magnetic resonance (MR) imaging, we looked for these possible specific subtypes of WM of distinct spatial distribution by a data driven approach. Each MR parametric map was considered as a kind of "staining" of the same tissue [7]. However, whereas classical staining is determined by the chemical properties of a tissue, MR parameter probe its biophysical properties. If different subtypes of WM have a different spatial distribution, they might be distinguished from one to another by different combinations of these MR "staining" contrasts. The combination of MR parameter values specific to one such subtype will be further referred to as "fingerprint".

Standard MRI is difficult to use for this purpose: it gives only a relative signal intensity, the signal is weighted by various MR parameters, and is biased by B_0_ and/or B_1_ field inhomogeneities and sometimes other hardware imperfections. Conversely, quantitative MRI allows the absolute measurement of selected MR parameters, e.g. R_1_ or R_2_, without bias from other instrumental or tissue parameters. In other words, it allows quantifying physical properties of a tissue. Beside R_1_ and R_2_, other physically independent parameters are of interest for WM. In our case, diffusion tensor imaging (DTI) was first used to calculate axial and radial diffusion (AD and RD). These two parameters are supposed to be related to tissue microstructure which has already been shown to be inhomogeneous [8,9]. Second, a gradient echo sequence was used to measure R_2_* from magnitude images which is especially sensitive to static local magnetic field inhomogeneity [10]. Phase images of the same sequence allowed to compute magnetic susceptibility (χ_m_), i.e. the extent to which a material is magnetized by an applied magnetic field [11]. In WM, χ_m_ is known to be negative, i.e. diamagnetic, in proportion to the myelin and its phospholipids content; conversely iron is paramagnetic and increase χ_m_ [11]. Last, MT imaging was used to work out the macromolecular bound proton fraction (MPF) which reflects the proportion of protons bound to macromolecules in a tissue. In WM this mostly reflects the amount of myelin [12–14].

In order to find spatially segregated WM voxels that might share the same fingerprint, spatial independent component analysis (sICA) was used [15]. sICA searched in every voxel the combination of independent component (IC) accounting for the observed distribution of tissue parameters. Each IC was a specific combination of parameters, i.e. a fingerprint, which linearly combines in one voxel to get to the observed tissue parameters and is supposed to be a subtype of WM. To define these IC, the method assumed that they were spatially independent from one another. However, "spatial independency" did not precluded that WM IC could partially overlap, i.e. a same voxel being a mix of different WM subtypes. The relative contribution of each parameter to the subtyping and the redundancy between them have been examined.

The differential effect of age and gender on the observed subtypes was also addressed. Some above-mentioned parameters were already known to vary in WM according to these variables. If one or both of these factors, which were not taken into account in this exploratory analysis, differently affected one or several parameter values of these WM subtypes, this would strengthen the validity of this decomposition. Such "external validators" are classically used in psychiatry as a proxy to validate the separation of different clinical categories [16]. We also explored in what respect this WM subtypes co-segregated with known WM fasciculi. The meaning of WM subtypes’ fingerprint will be discussed in terms of myelin content and constitution.

## Materials and methods

### Participants

This study received ethical approval from the "Comité de protection des personnes—Est IV", of Strasbourg (n°05/27 d). It has been performed in accordance with the Code of Ethics of the World Medical Association (Declaration of Helsinki). Twenty-six controls were recruited with the aim to represent at best a normal right-handed population: 12 women, age 38 ±9 Y, range 20–58 Y, years of education 13.8 ±2.5 Y, all right handed 90 ±9% according to the Edinburgh inventory [17]. Subjects were screened to exclude history of neurological or psychiatric disorders. Participants gave written informed consent and were remunerated for their participation.

### Imaging protocol

Participants took part in a single multi-parametric imaging session on a 3T Verio system (Siemens, Erlangen, Germany) with a 32-channel receiver head coil. Higher-order shimming was employed. An automated positioning and alignment of slices using anatomical landmarks (AAHScout) was used to ensure reproducible slice positioning from one subject to the other. First, a high-resolution 3D MP-RAGE image was performed: FOV = 224x224x157 mm^3^, matrix size = 320x320x224 (0.7 mm isotropic resolution), GRAPPA = 2, TR/TE/TI = 2400/2.41/1000 ms and flip angle θ = 8°. Acquisition scan time was 7 min 40 s.

Quantitative R_2_* and susceptibility mapping were then conducted using an axial 3D Multi Echo Gradient Echo (MGRE) sequence. Parameters were: FOV = 256×168×128 mm^3^, matrix size = 256×168×128, partial Fourier = 6/8. This protocol resulted in an isotropic resolution with a voxel size of 1×1×1 mm^3^. Parallel imaging (GRAPPA) reconstruction factor of 2 was used in the phase encoding direction (R-L) to speed up the acquisition (TR = 37 ms, TE1 = 2.21 ms up to TE8 = 28.11 ms with delta TE = 3.7 ms, monopolar read-outs, bandwidth = 400 Hz/px, θ = 20°). Acquisition scan time was 5 min 45 s.

R_1_ mapping was performed using the variable flip angle (VFA) method based on the spoiled 3D GRE (SPGR) sequence [18,19]: FOV = 240x240x160 mm^3^, matrix size = 192x192x128, i.e. 1.25 mm isotropic resolution, GRAPPA = 2. TR/TE = 20/2.25 ms and θ = 4 and 25°. Acquisition scan time was 7 min 30 s.

For quantitative MT mapping, a whole brain MT-weighted image was acquired using a sagittal 3D Gradient Echo (GRE) sequence (FOV = 240x240x160 mm^3^, matrix size = 192x192x128, i.e. 1.25 mm isotropic resolution, GRAPPA = 2, TR/TE1/TE2 = 28/2.25/6.90 ms and θ = 6°). Saturation MT pulse was a 12 ms Gaussian pulse, FA_MT_ = 560° and Δ = 6 kHz. Reference image (no-MT) for data normalization was obtained without saturation pulse. To increase signal-to-noise ratio, averaging of individual echo images was used for MT and reference image. For B_0_ mapping, we measured the phase difference between TE2 and TE1 MT images [20]. Acquisition scan time was 8 min 40 s.

R_2_ mapping was performed using the partially spoiled Steady State Free Precession (pSSFP) technique [21,22]. Imaging was performed in 3D (with sagittal orientation, H>>F for phase orientation) and with FOV = 240x240x180 mm^3^, matrix size = 192x192x144, i.e. 1.25mm isotropic resolution. Scans were performed with non-slice selective excitation pulses (300 μs duration) of 60° nominal flip angle and partial RF spoiling increments of 1 and 20 degrees (TR/TE = 7/3 ms and bandwidth = 500 Hz/Pixel). Acquisition scan time was 5 min 30 s.

Additionally, whole-brain 3D B_1_+ maps were acquired to correct for transmit field heterogeneities. B_1_+ maps were obtained using the actual flip-angle (AFI) imaging method based on a modified spoiled 3D GRE sequence (FOV = 260x260x180 mm^3^, matrix size = 48x48x36. FA = 60° (300 μs length hard pulses), TR2 = 5TR1 and TR1 + TR2 = 111 ms. TE = 2.75 ms, bandwidth = 240 Hz/pixel) [23]. Optimal spoiling of transverse relaxation was ensured by using an improved RF and gradient spoiling scheme as described in [24], assuming an isotropic scalar water diffusion coefficient D = 2.2 μm^2^/ms. Relevant parameters for spoiling were: diffusion damping = 0.300, RF spoil phase increment = 129.3°. Acquisition scan time was 3 min 13 s.

Whole brain DTI was conducted using a 2D RESOLVE sequence with TR/TE1/TE2 = 9400/83/108 ms, α = 90°, EPI factor = 55, bandwidth = 1136 Hz/Pixel, 20 gradient directions, and two b values of 0 and 1500 s/mm^2^. RESOLVE is based on a readout-segmented EPI strategy, allowing minimization of susceptibility distortions and T_2_* blurring [25]. Images were acquired with FOV = 220×220 mm, matrix = 110×110, in-plane resolution 2.0×2.0 mm^2^, slice thickness 2 mm, 64 slices, one signal average, and a scan time of 20 min 14 s.

Total scan time was about 60 minutes.

### Image preprocessing

All MP-RAGE were visually screened to exclude any anatomical abnormalities and T_2_ for white matter hyperintensities, i.e. ARWMC scale score = 0 [26]. All the computations were performed in MATLAB 12 (The MathWorks, Inc., Sherborn, MA, USA).

R_2_* maps were obtained with the 3D MGRE images acquired for QSM evaluation. The voxel-by-voxel R_2_* parameter was evaluated in a two-step procedure. Each R_2_* relaxation rate (in s^-1^) was calculated with a linear fit on the log-transformed data.

Quantitative susceptibility mapping (QSM) calculation was conducted from the phase of 3D MGRE images in STI Suite version 2.2 (http://people.duke.edu/~cl160/). Before QSM processing, complex images from multiple coils were combined in order to obtain a coherent phase image [27,28]. Mask images were generated from magnitude images by thresholding for background phase removal. Local phase evolution was estimated by a linear fitting of the phase images obtained at the 8 different TEs. A 3D-Laplacian operator was then used to perform phase unwrapping [29]. Background phase removal was performed using V-SHARP method [29]. Quantitative susceptibility mapping was then calculated from each local tissue phase by solving an inverse problem using the iLSQR method [30]. According to previous works [31,32] susceptibility values were not calibrated on CSF.

R_1_ (= 1/T_1_) was estimated voxel-wise by fitting the VFA-model equation [18] to experimental image data (Section A in S1 Text), with corrections for B_1_+ inhomogeneities. To account for insufficient spoiling of transverse magnetization, the correction described in [33] was applied.

MPF map was obtained using the single-point method [34]. MPF was estimated on a voxel-by-voxel basis by fitting MT-model equation (Eq. [1], Yarnykh, 2012, Section B in S1 Text) to experimental MT-weighted and reference image data, with corrections for B_0_ and B_1_+ inhomogeneities and constrained values of other model parameters: R_1f_ = R_1b_ = R_1_ = 1/T_1_, T_2b_ = 9.7μs, T_2f_ = 0.022 T_1_, k(1-f)/f = 19 cps.

R_2_ (= 1/T_2_) was derived from pSSFP image data, using eqs. [3] and [6] from [22] with corrections for B_1_+ inhomogeneities (Section C in S1 Text). A rough global T_1_ = 1.25 s was assumed for T_2_ computation [21,22]. T_2_ maps were thresholded at 150 ms. The relaxometric value R_2_’ (= R_2_*—R_2_) was introduced to specifically look for losses due to local field inhomogeneities [10,35].

AD, RD, ADC and FA were computed from the diffusion images after rigid registration [9]. Only AD and RD were used in the analysis in order to avoid dependence between the parametric maps. But ADC and FA could thus be extracted from the region of interest (ROI) of each subtype defined by the analysis.

All parametric images were spatially normalized using the MP-RAGE for parameter estimation using the Statistical Parametric Mapping toolbox 2012 (Welcome Department of Cognitive Neurology, London, UK). Images were generated without modulation by the Laplacian and resliced to the lowest resolution, i.e. 2 mm isotropic. Segmentation of WM and GM was performed on MP-RAGE images. Last, parameters maps, GM and WM images were mildly smoothed using a 5-mm isotropic kernel, as a compromise between the compensation of imperfection in the normalization process and the preservation of a reasonable resolution.

### Group Independent Component Analysis (ICA)

In order to give the same weight to each parameter map in the multivariate analysis, pictures from the different modalities were normalized in intensity. Since parametric maps gave absolute measurements, intensity normalization was performed at the group level in order to preserve inter-subject global variance over cohort. Accordingly, for each parameter, the group average and the group standard deviation of all in-brain voxels were used to scale the image between -4 to +4 times the standard deviations. These maximal values aimed at avoiding to give too much weight to possible outliers. Then all parameters’ images of all the participants were merged together, i.e. the 7 parametric images of each subject, in order to perform a group analysis (26 subjects x 7 parametric map—7 missing data = 175).

This 4D set was unfolded in order to get a 2D matrix by vectorizing the spatial dimension as input to the sICA. It was decomposed into N independent spatial maps using the INFOMAX algorithm as implemented in the FMRLAB software 2.3 (Swartz Center for Computational Neuroscience, University of San Diego, San Diego, CA, USA) [36].

As other ICA algorithm, INFOMAX is based on an iterative optimization function and thus its results might slightly depend on parameter initialization [37]. Moreover, there were no strong a priori regarding the number of components to extract [38]. Thus, to test for the consistency of the results, the same analysis was run for N = 20, 40, 60, 80, 100, 120 and 140 components.

To be retained as a WM subtype, the components had to fulfill two properties:

- To have a distribution of its positive component that spatially overlapped the WM.
- The component had to be shared by the subjects and should not be significantly influenced by the differences between them. That for we computed the ratio of between-parameters standard deviation over between-subjects standard deviation. It had to be above 1.5 for the component to be selected.

Reproducibility among the different decompositions using different number of components was evaluated using Fleiss’s kappa (κ) coefficient, which is a generalization of the Cohen’s kappa for cases with more than 2 judges [39]. WM subtypes of each analysis, i.e. for different extracted component, were thresholded and converted to binary maps which were considered as a judgment: WM subtype / not WM subtype. Fleiss κ was computed on these binary maps which gives us an evaluation of the reproducibility at the voxel level. The procedure was repeated for z ≥ 2 and z ≥ 3.2, the two thresholds used for display and parameter extraction respectively.

WM subtypes spatial map of each analysis performed at different extracted components were then averaged to define what would be further referred to as t-WM, f-WM and c-WM. The "xjView" function was used to display them using z > 2, k > 200 vx, i.e. 1.6 cm^3^ (http://www.alivelearn.net/xjview).

To get each component absolute values in their purest form, average fingerprints parameter values were extracted from the region of interest (ROI) defined by the average spatial component thresholded at a z-score ≥ 3.2. Moreover, to minimize the risk of getting mixed values due to the smoothing process, i.e. GM mixed up with WM values, only non-smoothed maps were used and were masked by the subject WM segmentation map to keep only voxels with a probability > 0.8 to be WM.

The contribution of each parameter in the difference between the subtypes was assessed using Cohen’s d. The cumulative sum of them gave an estimation of the distance between the subtypes and allowed to estimate the relative contribution of each parameter in their separation.

In order to see if some parameters were redundant, a cross-correlation was performed between them within each subtype. We used 2 exploratory thresholds uncorrected for multiple comparison: α = 0.5, i.e. r ≥ 0.38 for bilateral test, and α = 0.01, i.e. r ≥ 0.48.

The fingerprints of each WM subtype, i.e. their specific combination of parameter values, were represented on a radar plot together with the one of GM for comparison. Direct comparisons between their parameter values were performed using a paired t-test. All p-value below α = 0.05 will be displayed, but because of multiple testing, only p-values below p ≤ 0.011 should be considered as significant after correction for multiple comparisons (α = 0.05, 7x3 independent tests, Bonferroni method, i.e. family wise error rate correction).

The effect of WM subtype (within subject factor), age and gender, together with the interactions of WM subtype with age and gender, was assessed on each parameter using the generalized linear model (GLM) module of Statistica v.10 (StatSoft, Tulsa, OK, USA). We posit that the decomposition would be further legitimated if one interaction with an external validator was significant at α = 0.05 for the global analysis. For parameter-wise analysis, although all p-value below α = 0.05 will be displayed, because of multiple testing, only p-values below p ≤ 0.019 should be considered as significant after correction for multiple comparisons (α = 0.05, 7 independent tests, Bonferroni method, i.e. family wise error rate correction).

Last, superposition with known WM fasciculi was achieved using the average white-matter tractography atlas from 10 adults from Johns Hopkins University [40] of the anterior thalamic radiations, the corticospinal tract, the inferior fronto-occipital fasciculus, the superior and inferior longitudinal fasciculus, the uncinate fasciculus, frontal and occipital forceps of the corpus callosum. The proportion of track superposition with each WM subtype was computed using a more permissive threshold of z > 2.

## Results

For technical reasons, data were missing. Susceptibility, R_2_* and MPF maps were missing for 2 subjects, R_1_ map for one, and R_2_ map for another one.

### Three white matter subtypes

Three components fulfilled the above-mentioned criteria. These three WM subtypes will be further referred to as t-WM, f-WM and c-WM for track-, frontal- and central-WM according to their spatial distribution (see later). The reproducible partitioning in 3 WM components was obtained for all ICA decompositions when the number of components to be extracted was set between 60 and 120, tested on every tens. κ values for t-WM were 0.85 and 0.88 (for z ≥ 2 and 3.2 respectively), f-WM had 0.70 and 0.72 and c-WM 0.66 and 0.64, which range from substantial to perfect agreement at the voxel level on the 4 measurements.

**Fig 1:**
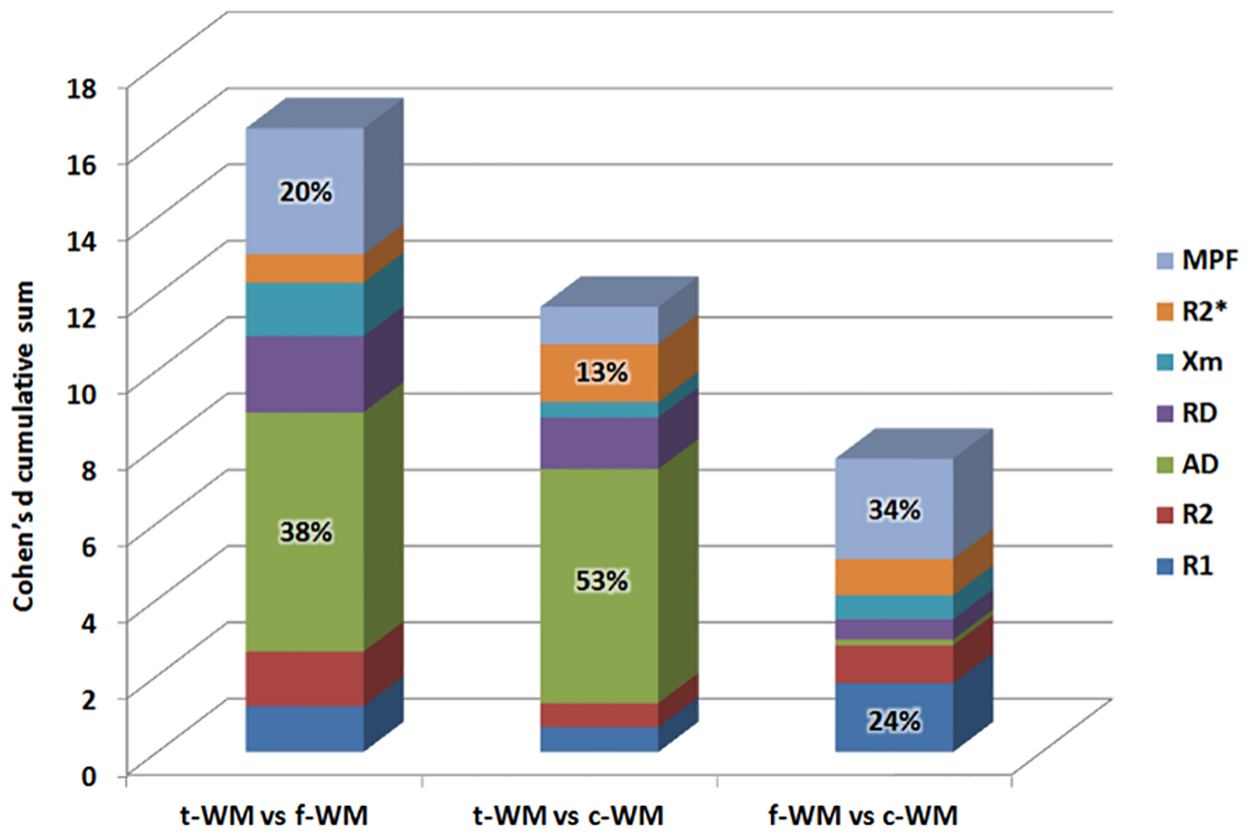
Contribution of the different parameters in separating the subtypes. For each pairs of subtypes, the bars represent the distance between the subtypes expressed in the cumulative sums of Cohen’s d. The contribution of each parameter is shown in different color. t-WM showed the largest absolute difference with f- and c-WM essentially because of large differences in the AD parameter. However, it was virtually of no value to separate f-WM from c-WM. MPF was also a substantial contributor to subtyping, especially between f-WM from c-WM.

For a number of extracted components between 60 and 120, t-WM explained 4.9 ±0.4% of the total variance, i.e. all voxels included in the analysis, f-WM: 8.3 ±1% and c-WM: 2.4 ±0.4%. This made them in the first ten components explaining the largest amount of variance. The inter-parameter/inter-subject ratios were 4.8 ±2 for t-WM, 6.7 ±2 for f-WM and 2.5 ±0.7 for c-WM, overly corresponding to a population outcome. The contribution of each parameters to the separation in the different subtypes is presented in Fig 1. The average relative contribution of each parameter was between 10–12% except two larger ones for AD and MPF (31% and 21% respectively) and a smaller one for χ_m_ (7%). Regarding the cross-correlation tables, there were no overwhelming redundancy although AD and RD, and RD and R2* were consistently correlated (see Table 1).

**Table 1:** Correlation matrix between the parameters within each WM subtypes.

**t-WM**	**R1**	**R2**	**AD**	**RD**	**Xm**	R2*
**R2**	-0.05					
**AD**	-0.21	-0.14				
**RD**	-0.19	-0.20	**0.70**			
**Xm**	0.19	-0.17	-0.02	0.10		
R2*	0.11	0.32	-0.40	**-0.65**	-0.36	
**MPF**	0.10	0.44	-0.19	**-0.50**	-0.34	**0.54**
**f-WM**	**R1**	**R2**	**AD**	**RD**	**Xm**	R2*
**R2**	-0.21					
**AD**	0.29	0.02				
**RD**	-0.03	-0.11	**0.50**			
**Xm**	0.32	0.02	-0.20	-0.06		
R2*	0.00	0.37	-0.42	**-0.49**	-0.28	
**MPF**	0.34	0.26	0.23	**-0.53**	-0.27	0.23
**c-WM**	**R1**	**R2**	**AD**	**RD**	**Xm**	R2*
**R2**	**-0.53**					
**AD**	-0.21	0.03				
**RD**	-0.11	-0.04	**0.63**			
**Xm**	0.01	-0.18	0.05	-0.27		
R2*	0.02	0.36	**-0.48**	**-0.53**	0.08	
**MPF**	-0.27	0.42	-0.08	-0.28	-0.09	0.12

Significant values at α = 0.5, i.e. r ≥ 0.38 for bilateral test are in non-shaded boxes, and α = 0.01, i.e. r ≥ 0.48 are shown in black bold font.

**Fig 2:**
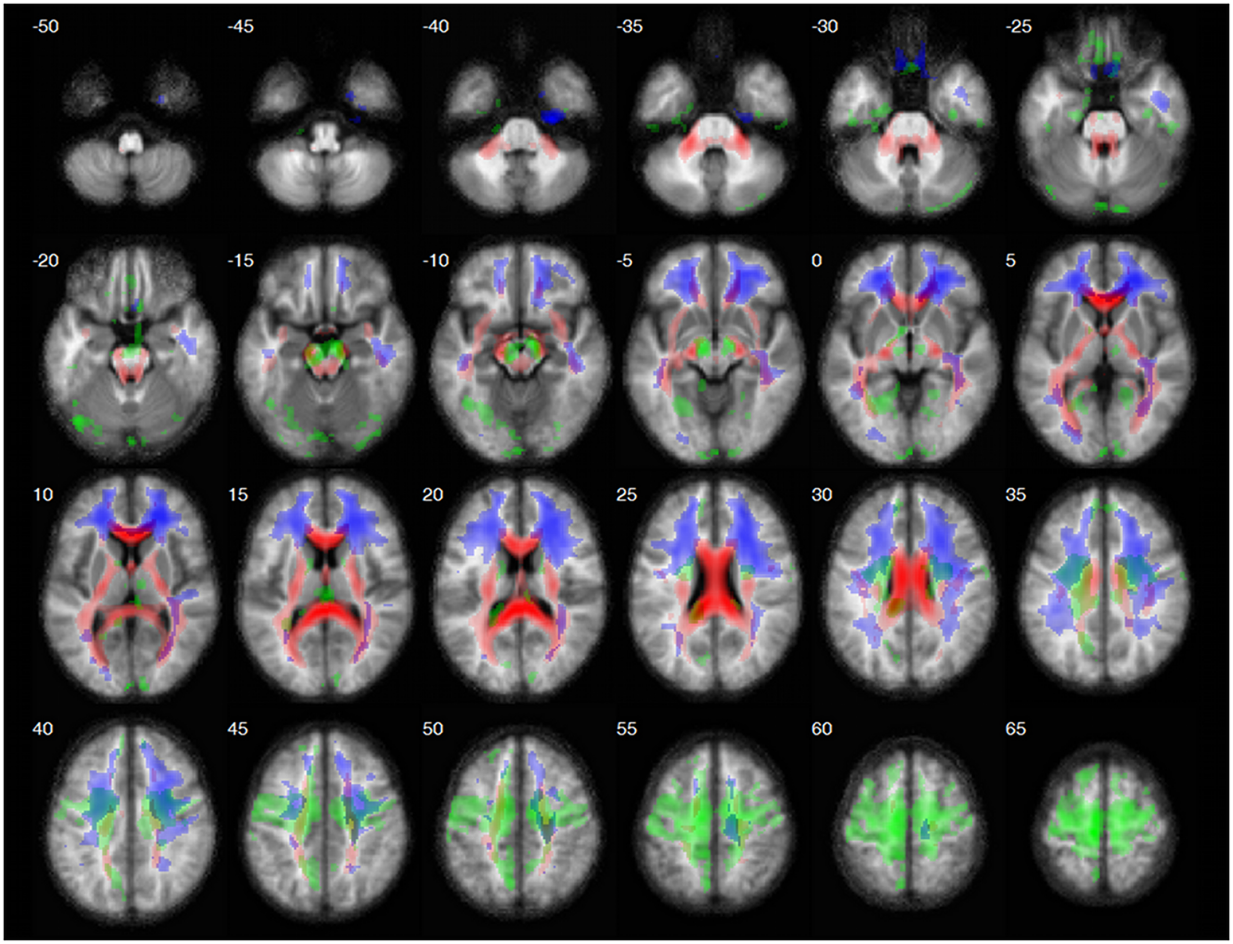
Spatial distribution of the average t-WM (red), f-WM (blue) and c-WM (green). Positive components are displayed on the average MPF map (not smoothed). z ≥ 2, k ≥ 200 vx (1.6 cm^3^). t-WM encompassed deep WM regions of well-structured tracts: the corpus callosum, the extreme, external and internal capsules, the corona radiata, the cerebellar peduncles (superior, middle and inferior), the pons and the mesencephalon. f-WM and c-WM mainly corresponded to subcortical WM regions: the centrum semiovale including U-fibers in some places. f-WM was mostly frontal while c-WM was essentially central corresponding to the cortico-bulbar and cortico-spinal tracts.

The spatial distribution of t-WM (in red, Fig 2) clearly mapped on well-formed parts of projection and commissural tracts in deep WM, e.g. corpus callosum, corona radiata, extreme, external and internal capsule. Conversely f-WM (in blue) overlapped with superficial or subcortical WM mainly corresponding to association tracts in the centrum semiovale and was especially prominent in the frontal lobes. Last, c-WM (in green) corresponded to subcortical WM arising from the central cortex which essentially mapped the cortico-bulbar and cortico-spinal tract. All subcomponents were essentially symmetrical and did only marginally overlap with one another. Full volume of z-maps are available for download (http://www.cercle-d-excellence-psy.org/fileadmin/cep_files/Neurocrypto/3mapsWM.zip and has a DOI 10.6084/m9.figshare.5946781).

### Fingerprints of white matter subtypes

**Fig 3:**
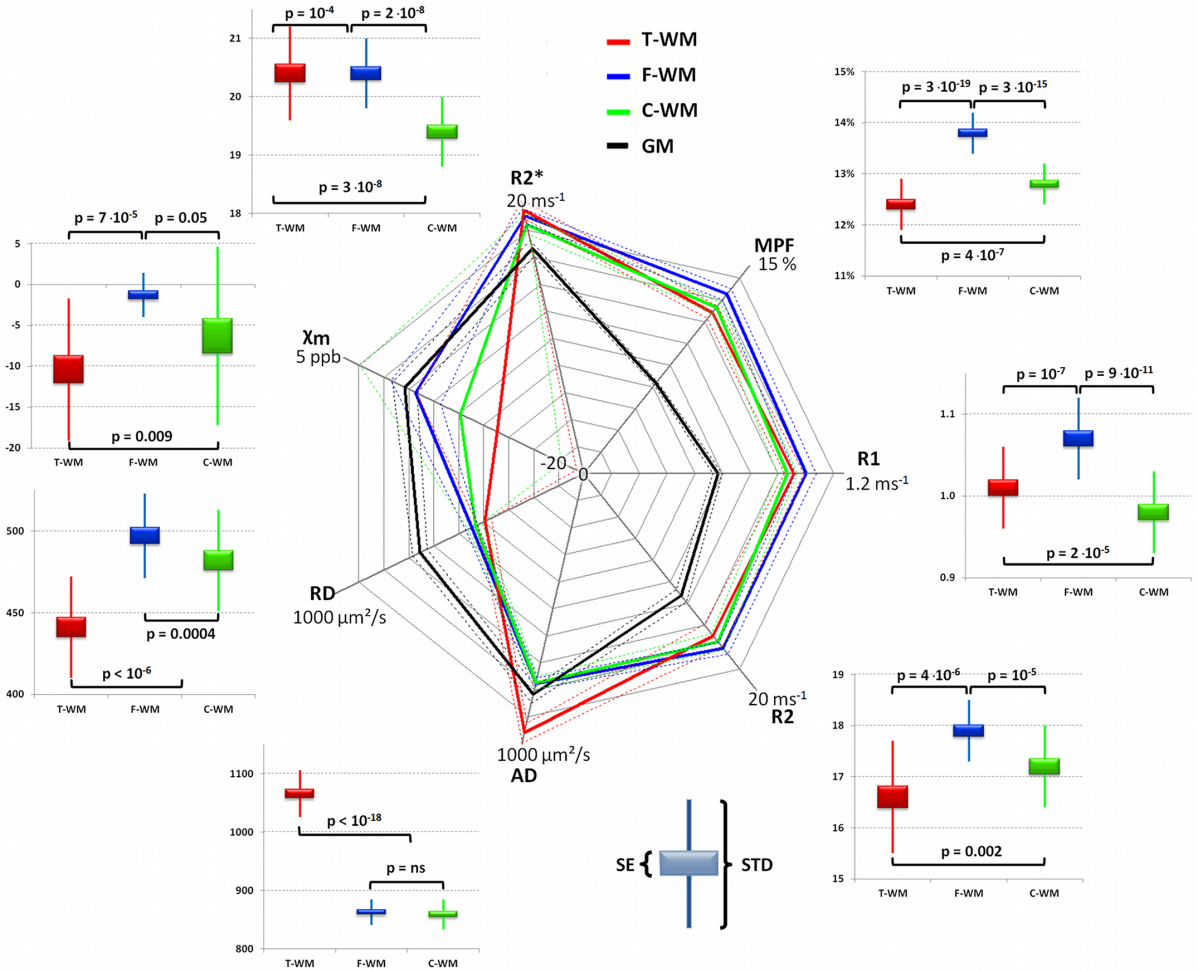
Fingerprints of each WM subtypes and GM. The "absolute values" of population average are shown in thick lines and standard deviation in dotted lines on the polar plot in the middle. Center is 0 except for susceptibility (-20 ppb) and maximal values are displayed on each axis. t-WM is shown in red, f-WM in blue, c-WM in green and GM in shown as a comparator in black. Boxplots around the central graph give the standard error (SE—solid box) and the standard deviation (SD—whiskers) of each WM subtype according to the parameter.

The fingerprints of each WM subtypes looked very similar in comparison with GM fingerprint except on diffusion and susceptibility (see radar plot in Fig 3). Nevertheless, the differences between WM subtypes were very significant on nearly all parameters (see Table 2). t-WM had the largest AD, ADC, FA, R_2_* and R_2_’ values. f-WM had the largest R_1_, R_2_, RD, χ_m_ and MPF values, it was also more likely to be recognized as WM according to SPM segmentation. t-WM had larger R_1_, AD, FA, R_2_*, R_2_’ and MPF values than c-WM, whereas the latter had larger RD values, it was also less likely to be classified as WM. Last, f-WM had larger R_1_, R_2_, R_2_*, MPF and FA values than c-WM.

**Table 2:** Parameter values of WM subtypes.

	Absolute measurements			Comparisons (p-values)			Order
	t-WM	c-WM	c-WM	t vs. f	t vs c	f vs c	
**R1**	1.01 ±0.05	1.07 ±0.05	0.98 ±0.05	1.0 ∙10^−7^	1.7 ∙10^−5^	9.0 ∙10^−11^	f-WM > t-WM > c-WM
**R2**	16.6 ±1.1	17.9 ±0.6	17.2 ±0.8	4.4 ∙10^−6^	0.002	1.0 ∙10^−5^	f-WM > c-WM > t-WM
**AD**	1066 ±40	863 ±22	859 ±26	7.0 ∙10^−20^	2.4 ∙10^−19^	ns	t-WM > f-WM = c-WM
**RD**	441 ±31	497 ±26	482 ±31	2.0 ∙10^−10^	1.2 ∙10^−6^	4.1 ∙10^−4^	f-WM > c-WM > t-WM
**Xm**	-10.4 ±8.7	-1.3 ±2.7	-6.3 ±10.9	6.9 ∙10^−5^	0.009	0.048	f-WM ≥ c-WM > t-WM
R2*	21.7 ±0.8	21.1 ±0.6	20.4 ±0.8	1.0 ∙10^−4^	2.7 ∙10^−8^	1.6 ∙10^−8^	t-WM > f-WM > c-WM
**MPF**	12.4 ±0.5	13.8 ±0.4	12.8 ±0.4	3.5 ∙10^−19^	3.7 ∙10^−7^	3.2 ∙10^−15^	f-WM > c-WM > t-WM
**ADC**	668 ±36	625 ±23	614 ±28	7.1 ∙10^−7^	5.8 ∙10^−8^	5.4 ∙10^−4^	t-WM > f-WM > c-WM
**FA**	0.52 ±0.02	0.39 ±0.02	0.40 ±0.02	8.2 ∙10^−24^	6.3 ∙10^−21^	3.1 ∙10^−5^	t-WM > c-WM > f-WM
**R2’**	5.0 ±1.2	3.2 ±0.7	3.2 ±0.9	9.5 ∙10^−7^	9.5 ∙10^−9^	ns	t-WM > f-WM = c-WM
**pWM**	99% ±0.1	100% ±0.1	98% ±0.2	3.0 ∙10^−21^	9.3 ∙10^−18^	4.5 ∙10^−23^	f-WM > t-WM > c-WM

Population averages ± standard deviations of the parameters were computed from the subjects average within the ROIs defined by the average component maps, i.e. t-WM, f-WM and c-WM thresholded at z = 3.2 (see text). R1, R2, R2* and R2’ are expressed ms^-1^, AD, RD and ADC in μm^2^/sec, volume susceptibility χ_m_ is given in ppb (part per billion), MPF is in percentage of bound protons, FA is a fraction, and the probability of WM (pWM) is expressed in percentage. The p-values refer to the paired t-test t-WM and f-WM, t-WM and c-WM and f-WM and c-WM.

### The effect of age and gender on the three WM subtypes

The effects of age and gender on the parameters of the different WM subtypes are given in Table 3. Not only age had a global effect, i.e. an effect that was independent of the WM type, (F(7,13) = 4.1, p = 0.014) but it also had a differential effect depending on the specific WM type, i.e. an interaction between age and WM type (F(14,6) = 4.7, p = 0.033). There was no gender effect on the parameters selected for the analysis.

**Table 3:** Global and specific effect of age and gender on WM subtypes.

	**Omnibus**													
	***F-value***	***p-value***												
**Age**	F(7,13) = 4.1	0.014												
**Gender**	F(7,13) = 0.6	ns												
**Type**	F(14,6) = 891	9.3 ∙10^−9^												
**Type x Age**	F(14,6) = 4.7	0.033												
**Type x Gender**	F(14,6) = 0.9	ns												
	**R1**		**R2**		**AD**		**RD**		**Xm**		R2*		**MPF**	
	***F-value***	***p-value***	***F-value***	***p-value***	***F-value***	***p-value***	***F-value***	***p-value***	***F-value***	***p-value***	***F-value***	***p-value***	***F-value***	***p-value***
**Age**	F(1,22) = 0	ns	3.7	ns	0.8	ns	2.7	ns	0.7	ns	0.0	ns	10.6	0.004
**Gender**	F(1,22) = 2.3	ns	0.1	ns	3.5	ns	1.2	ns	0.2	ns	0.1	ns	0.6	ns
**Type**	F(2,22) = 31.4	3.4 ∙10^−9^	0.2	ns	21.6	2.4 ∙10^−7^	7.5	0.001	2.4	ns	12.7	4.5 ∙10^−5^	26.3	4.0 ∙10^−8^
**Type x Age**	F(2,22) = 11.2	1.2 ∙10^−4^	1.1	ns	2.7	ns	1.4	ns	1.5	ns	3.3	0.047	1.9	ns
**Type x Gender**	F(2,22) = 0.2	ns	0.4	ns	1.9	ns	1.6	ns	0.1	ns	0.8	ns	0.1	ns
	**ADC**		**FA**		**R2’**									
	***F-value***	***p-value***	***F-value***	***p-value***	***F-value***	***p-value***								
**Age**	F(1,23) = 2.6	ns	9.2	0.006	2.1	ns								
**Gender**	F(1,23) = 5.2	0.031	0.6	ns	0.1	ns								
**Type**	F(2,23) = 0.1	ns	46.2	1.0 ∙10^−11^	2.6	ns								
**Type x Age**	F(2,23) = 2.7	ns	2.2	ns	0.6	ns								
**Type x Gender**	F(2,23) = 3.6	0.036	0.6	ns	0.7	ns								

First row is the GLM omnibus test which took into account all the parameters used in the decomposition, i.e. the one of the second row. Second row gives the result of the analysis for each of these parameters independently. On the last row, the same analysis was performed on the parameters that were not used in the decomposition. Global effect, i.e. effect that affected all WM subtypes, are in the white rows. Specific effects were assessed by WM subtype x age and gender interaction (lower rows in gray). ns: non-significant.

**Fig 4:**
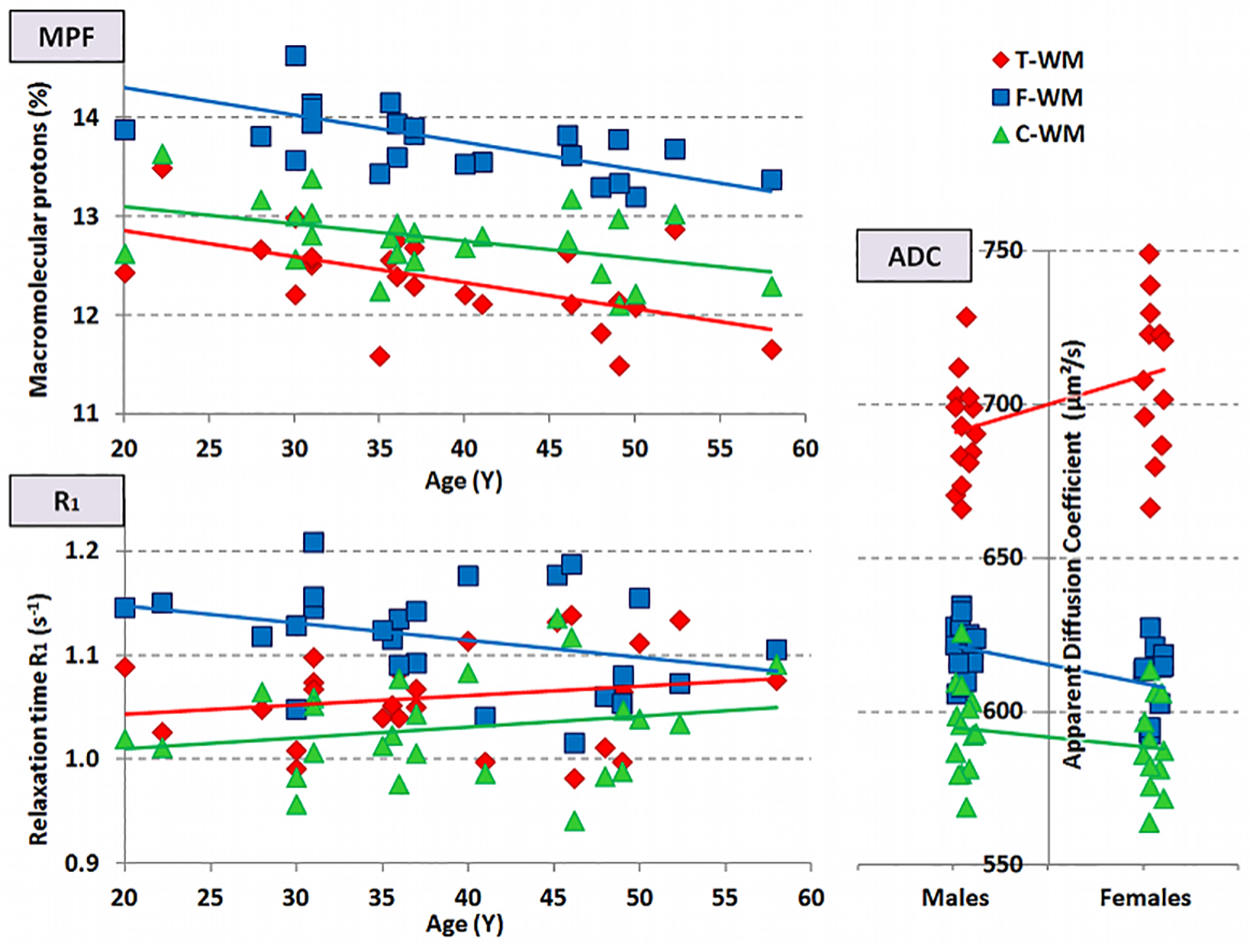
Age and gender effect on MPF, R_1_, and ADC. **a.** Upper left graph: Age effect on MPF. All subtypes showed a similar decrease with aging (F(1,22) = 10.6, p = 4 10^−3^, no interaction). **b.** Lower left graph: Age effect on R_1_. Whereas R_1_ decreased with aging in f-WM as for MPF, it increased in t-WM and c-WM (f-WM vs. t-WM: F(1,22) = 16.6, p = 5.0 10^−4^; f-WM vs. c-WM: F(1,22) = 15.9, p = 6.2 10^−4^). There was a differential effect of aging on MPF and R1 in t-WM and c-WM only. **c.** Right graph: Gender effect on ADC. Gender had a selective effect on t-WM with females having larger ADC than males, whereas it had virtually no effect on f-WM and c-WM (t-WM vs. f-WM: F(1,23) = 5.7, p = 0.025; t-WM vs. c-WM: F(1,23) = 2.3, ns).

Age had a global effect on MPF showing decreasing values with increasing age (see Fig 4a, F(1,21) = 10.6, p = 3.8 10^−3^). FA, which was not part of the original analysis, also came with a significant age-related reduction (F(1,23) = 9.2, p = 0.006).

Specific effect of age, i.e. significant WM subtype x age interaction, was significant on R_1_ and R_2_* (F(2,22) = 11.2, p = 1.2 10^−4^ and F(1,22) = 3.3, p = 0.047 respectively). Whereas R_1_ decreased from 20 to 58 years in f-WM, it increased with aging in t-WM and c-WM (see Fig 4b, f-WM vs. t-WM: F(1,22) = 16.6, p = 5.0 10^−4^; f-WM vs. c-WM: F(1,22) = 15.9, p = 6.2 10^−4^). R_2_* decreased with aging from 20 to 58 years in t-WM, increased in c-WM while it remained stable in f-WM (see Fig 4b—t-WM vs. c-WM: F(1,22) = 7.9, p = 0.010).

Although not part of the original analysis, ADC was affected by gender as a general effect and a specific effect. Average diffusivity was larger in women (F(1,23) = 5.2, p = 0.031). But gender interacted also with WM type (F(2,23) = 3.5, p = 0.036) with t-WM having larger ADC values in women than men whereas this factor had impact neither on f-WM (WM1 vs. f-WM: F(1,23) = 5.7, p = 0.025) nor on c-WM (ns) (see Fig 4c).

### Co-segregation of WM subtypes with classical fasciculi

Strikingly, the spatial distribution of WM subtype did not segregate with fasciculi (see Table 4 and Fig 2). The pyramidal tracts for example first ran through c-WM subpart just below the cortex before getting through t-WM at the level of the corona radiata up to the pons. Similarly, most anterior callosal fibers were part of t-WM while passing in f-WM areas when approaching the cortex. There were however exceptions to this rule, e.g. most fibers in the occipital forceps of the corpus callosum remained within t-WM.

**Table 4:** Proportion of WM subtype in the main fasciculi.

Track	L/R	t-WM (%)	f-WM (%)	c-WM (%)
Antenior Thalamic Radiation	R	22%	37%	11%
	L	24%	36%	10%
Corticospinal Tract	R	58%	1%	50%
	L	59%	18%	41%
Inferior Fronto-occipital Fasciculus	R	54%	35%	6%
	L	45%	55%	1%
Inferior Longitudinal Fasciculus	R	45%	15%	9%
	L	35%	32%	1%
Superior Longitudinal Fasciculus	R	19%	37%	15%
	L	18%	35%	9%
Uncinate Fasciculus	R	45%	25%	0%
	L	35%	52%	1%
Corpus callosum—Frontal forceps		28%	57%	1%
Corpus callosum—Occipital forceps		62%	8%	3%

The inferior fronto-occipital, the inferior longitudinal, and the uncinate fasciculi had balanced belonging to t-WM and f-WM. The anterior thalamic radiation and the superior longitudinal fasciculi were more mixed with f-WM predominance while the cortico-spinal tract was balanced between t-WM and c-WM. The case of the transcallosal fibers was very different between the frontal forceps (mainly f-WM) and the occipital one (mostly t-WM).

## Discussion

The sICA revealed very consistent components which only marginally spatially overlapped with one another. These spatially congruent WM areas had very reproducible fingerprints from one subject to another, such that their parameter values were significantly different. Moreover, some WM subtypes also varied differently according to gender and age on specific parameters, further suggesting that they might be of distinct kinds. Intriguingly, their spatial distribution did not segregate with fasciculi, but fibers from the same fasciculi could run generally through at least two different subtypes.

### Interpretation of the different WM fingerprints

Regarding the diffusion parameters, t-WM had the larger AD, ADC, but also the larger FA which is compatible with well-organized WM bundles [9]. Accordingly it mapped with well-organized part of known fasciculi. In contrast, both subcortical WM subtypes, the frontal f-WM, and the central c-WM were located in regions where fibers cross and where the tracks might be mixed with one another including U-fibers at least underneath the central cortex. Their higher RD might be best interpreted as fiber crossing rather than reduced myelination [8]. Both had similar AD but c-WM had a higher FA and a smaller RD which could be interpreted as a more organized section of subcortical WM mainly composed of cortico bulbar or cortico-spinal tracts.

MPF gives the fraction of macromolecular protons in a voxel [34]. Although, myelin macromolecules also consist in proteins [41], lipids seem to be responsible for most of the magnetization transfer contrast [12]. Whatsoever, MPF increases in proportion to the amount of myelin [42]. Thus both f-WM and c-WM had more myelin relative to t-WM’s organized WM tracts according to their higher MPF. This is consistent with ex-vivo measurements in humans, showing 12% more myelin per gram of WM in the frontal WM (f-WM) than the callosal WM (t-WM) [6].

In WM, susceptibility is primarily determined by two constituents which have opposite effects. First, the phospholipids of the myelin have a mild diamagnetic property concordant with the negative χ_m_ values of each WM subtypes [11]. Second, the iron atoms unbound to oxygen have a strong paramagnetic property [11] which partially counterbalance the diamagnetism of phospholipids and explains most of the differences in χ_m_. The higher χ_m_ values of f-WM fits well with the ex-vivo measurements of higher iron content in U-fibers which are specially prominent in the frontal WM [4]. Brain’s iron storage proteins, i.e. ferritin and transferrin, are known to be primary stored in oligodendrocytes, the cells that makes myelin [43]. Thus this result suggest that f-WM might contain either more oligodendrocytes or oligodendrocytes with higher iron content. Considering that iron content is supposed to be proportional to the metabolism needed to maintain myelin membranes [44], higher χ_m_ values of f-WM are in line with a richest myelin content in agreement with its higher MPF values. c-WM values might be interpreted similarly, having an intermediate status between f-WM and t-WM.

Longitudinal relaxation R_1_ is an energy dissipative process by which protons’ spins having been put in a high energy state by the RF pulse will return to their low energy state. Myelin-rich and/or iron-rich surroundings are known to speed up the process, i.e. to increase R_1_ [45]. f-WM had the largest R_1_ which again points toward its higher myelin content and its higher iron content. However, t-WM had a significantly larger R_1_ value than c-WM which point toward the opposite direction relative to MPF and χ_m_ results. Thus we might consider other factors such as the chemical constitution of the myelin. Considering its lipid component, R_1_ is especially sensitive to its cholesterol and galactocerebroside content [13,46]. Although MT, and thus MPF, seems to be impacted in the same proportion by galactocerebroside [13], the impact of cholesterol appears to be smaller on this parameter [14] which makes it a good candidate to explain this MPF / R_1_ discrepancy. Considering that cholesterol represent about a third of the myelin lipids and that its molar fraction, i.e. cholesterol / phospholipids, is 20% larger in t-WM regions, i.e. brain stem and cerebellum, than in c-WM regions, i.e. semioval center [5], this could well account for the larger R_1_ in t-WM.

R_2_ relaxation rate refers to transverse magnetization dephasing which is a pure entropic process without energy loss. R_2_ is affected both by macromolecule as it increases with increased myelination [13]. R_2_ has also been showed to be closely correlated with brain iron content although the correlation seemed to be weaker for WM than for GM [47]. Since we observed the same ordering for R_2_ values than for MPF and χ_m_ values, this strengthens the idea of increasing macromolecular content from t-WM, c-WM to f-WM and/or oligodendrocytic iron content from t-WM, c-WM to f-WM.

R_2_* relaxation shares all R_2_ factors but adds an important one: the role of inhomogeneous static local magnetic field. This factor played a sufficiently prominent role for R_2_* and R_2_ not to be significantly correlated. The specific contribution of static local magnetic field is captured by the parameter R_2_’ which is the difference between R_2_* and R_2_ [10]. Paramagnetic atoms such as iron are generally considered to play a fundamental role [47]. But this does not fit with the present observations as t-WM had the highest R_2_* and R_2_’ values. These higher R_2_* values have already been reported to be unrelated to iron concentration in WM bundle such as the corpus callosum and optical radiations [1]. Indeed t-WM’s lowest χ_m_ values might well explain this observation: as t-WM had the largest absolute χ_m_ value, its static local magnetic field might be more inhomogeneous than in f-WM and c-WM. This could well account for the largest R_2_’ of this subtype. Thus, in WM, the nature of this R_2_’ effect might be related to the diamagnetic property of the myelin sheath [11], uncompensated by the paramagnetic effect of iron.

### The contrasting effect of age and gender on WM subtypes

For all WM subtypes, FA values decreased with increasing age in line with previous literature [48]. As neither AD nor RD were significant, both a slight decrease in AD and an increase in RD contributed to the decrease in FA. Similarly, MPF values decreased with aging in accordance with a previous observation [49]. There was no difference between subtypes. Although age has been reported to impact R_1_ [50], R_2_ [51] and ADC [52], these were non-significant in our analysis. The present failure to detect age-related changes might be related to the WM regions where age-related changes were assessed. The significant WM subtype x age interaction showed that R_1_ reduction could only be observed in f-WM regions. Thus R_1_ and MPF pointed toward a myelin loss in f-WM, but there was again a MPF / R_1_ dissociation regarding the age effect in t-WM and c-WM. This could fit with the ex-vivo measurement of cholesterol fraction in human brains, which has been described to increase with aging [53].

Although not part of the original analysis, ADC was affected by gender as a general effect and a specific effect. Average diffusivity was larger in women, an effect that has been inconsistently reported in the literature [54,55]. But this effect was even larger for t-WM. Interestingly this interaction could explain the above-mentioned inconsistencies between studies as the gender effect appears to depend on the WM subtype.

### Significance of WM subtypes

t-WM appeared to be specific to WM implicated in well-organized fasciculi (AD, FA). This subtype was however the less rich in myelin (MPF), which replicates previous observations of poor correlation between WM organization and its macromolecular content [42]. According to its lowest iron content (χ_m_), it might also be the less metabolically active subtype, having the lowest membrane turnover. According to its position in core pathways, it makes sense for t-WM to be the more stable subtype. This "organized and stable myelin" might also be richer in cholesterol (MPF / R_1_ discrepancy) which also makes sense as cholesterol promotes closer membrane to membrane contact and allows myelin to be more tightly packed [56] together as increasing its electrical insulating role [57].

f-WM was the less organized at a macroscopic level (AD, FA), but the richest subtype in myelin (MPF). According to its highest iron content (χ_m_), it might also be the most metabolically active which might go with a high membrane turnover. The latter might include cholesterol synthesis (R_1_) as myelin cholesterol is quite entirely synthesized de novo by the oligodendrocyte and is energetically costly [57]. This "frontal subcortical WM" was dense but changing, i.e. adaptive, in line with the fact that they are the latest to mature and are supposed to be important in the development of higher cognitive functions [58].

Last, c-WM shared some similarity with f-WM. This "central subcortical WM", was also poorly organized (AD) but more intermediate in its myelin richness (MPF) and its iron content (χ_m_), which might point toward a slightly lower membrane turnover. It might be poorer in cholesterol (MPF / R_1_ dissociation) even though this might increase with age.

Although spatially independent "in essence", WM subtypes slightly overlap, probably not only due to the image smoothing. This suggests that oligodendrocytes from one or another subtype might share the same space. Thus, there might be no border but rather a smooth gradation between one subtype to another explaining why high-resolution WM explorations did not report them already.

Strikingly, the spatial distribution of the WM subtypes did not segregate with fasciculi. Thus, myelination subtypes appeared to change along the tracts. Within the cortex, using electron microscope reconstruction of single axons, myelination has already been shown to be non-uniform along the length of axons [59]. This has led to the assumption that myelination of one axon was not an all-or-nothing phenomenon but rather dependent on the surrounding oligodendrocytes. The present results suggest that this might also be true within white matter areas where the axon might be more tightly wrapped with dense and stable myelin and within other areas with less packed, but richer and more rapidly renewed or changing myelin.

Limitations should be considered however. First as these results were mainly derived from an exploratory analysis, they need to be replicated in an independent cohort. This should be especially considered when studying development or pathologies. Moreover, the decomposition might be dependent on the parameter maps used as input. Here, AD played a prominent role in separating t-WM from f-WM and c-WM, but it was virtually of no value to separate f-WM from c-WM. MPF was also a substantial contributor to subtyping, especially between f-WM from c-WM. However, all other parameters contributed for at least 10% on average, except χ_m_ which average contribution was of 7% and the redundancy between them was limited. Yet it is probable that including other parameters such as proton density [60], multi-compartment relaxometry [61], neurite density and orientation dispersion index [62] and perhaps even conductivity and permittivity [63], might further refine WM subtyping. Our "external validators" can only be considered as poor proxies for the validity of these subtypes. Appropriate validation might come from the use of high resolution WM explorations.

Last, it has been estimated that one 2x2x2 mm voxel contains between 0.5 to 5 million axons, and about 0.7 million oligodendrocytes [64]. Accordingly, the above MRI measurements only gave an average for each voxel whereas within the voxels, the discrepancies between association fibers and projection fibers remain. The disentangling of these two hypotheses will need histological approaches.

## Conclusions

Multi-parametric quantitative MRI allowed to separate three subtypes of WM. t-WM, consisted in the most compact and structured parts of projection and commissural tracts. It was less myelinated and less renewed but with a myelin that might be richer in cholesterol, more compact and isolating. Conversely f-WM, the frontal subcortical WM, was less macroscopically structured, but the richest in myelin and its iron richness pointed toward a high membrane turnover which might favor plasticity. c-WM, the central subcortical WM, shared many properties with the frontal subtype, although it was less prone to renewal and possibly less rich in cholesterol. Although age had a general impact on myelination, the structured and the central subcortical WM might increase their cholesterol content which might not be the case for the frontal subtype.

The quantitative nature of these measures can potentially allow inter-scanner reproducibility and inter-subject comparison for developmental and pathological studies. Although next move should be to replicate these results, it opens the perspective to refine our understanding of WM in development and pathologies not as a single entity but according to different subtypes.

## Supporting information

S1 Text Image processing equations for: A) T1 mapping by VFA method; B) MPF mapping by single-point method; C) T2 mapping using the T2-pSSFP method.s.
(PDF)
