# Supplementary material for: Multi-parametric quantitative MRI reveals three different white matter subtypes
Source: PLoS One. 2018 Jun 15;13(6):e0196297. doi: 10.1371/journal.pone.0196297 (PMC6003690; doi:10.1371/journal.pone.0196297)
Supplement: S1 Text — Image processing equations for: A) T1 mapping by VFA method; B) MPF mapping by single-point method; C) T2 mapping using the T2-pSSFP method.s. (PDF) [file pone.0196297.s001.pdf]

Annexe I – Image processing equations

A. T1 mapping (VFA method)

The signal amplitude S_i in the pulsed steady state derived for a spoiled gradient-echo (SPGR) sequence acquired at a flip angle α_i is a function of the longitudinal relaxation time T_1, repetition time TR, and equilibrium magnetization M0:

$$S_i = M_0 \sin\alpha_i \frac{1 - E_1}{1 - \cos\alpha_i E_1}$$

where $E_1 = \exp(-TR/T_1)$.

B. MPF mapping (single-point method)

The longitudinal magnetization in the pulsed steady state derived for a SPGR sequence with off-resonance preparative pulse based on the two-pool MT theory can be expressed in matrix form as follows:

$$\boldsymbol{M}_z = (\boldsymbol{I} - \boldsymbol{E}_s\boldsymbol{E}_m\boldsymbol{E}_r\boldsymbol{C})^{-1}\{[\boldsymbol{E}_s\boldsymbol{E}_m(\boldsymbol{I} - \boldsymbol{E}_r) + (\boldsymbol{I} - \boldsymbol{E}_s)]\boldsymbol{M}_{eq} + \boldsymbol{E}_s(\boldsymbol{I} - \boldsymbol{E}_m)\boldsymbol{M}_{ss}\},$$

where $\boldsymbol{M}_z$ is the vector with components M_z^F and M_z^B corresponding to the longitudinal magnetization of the free and bound pool immediately before the excitation pulse; $\boldsymbol{M}_{eq}$ is the vector of equilibrium magnetization with elements $M_0(1-f)$ and $M_0 f$, where f is MPF; $\boldsymbol{M}_{ss}$ is the vector of steady-state longitudinal magnetization. $\boldsymbol{I}$ is the unit matrix. The terms $\boldsymbol{E}_s$ and $\boldsymbol{E}_r$ describe longitudinal relaxation during delays before and after the excitation pulse. The matrix term $\boldsymbol{E}_m$ describes off-resonance saturation by the MT pulse. Explicit notation for $\boldsymbol{M}_{ss}$, $\boldsymbol{E}_s$, $\boldsymbol{E}_m$, $\boldsymbol{E}_r$ and can be found elsewhere (Yarnykh , 2012; Yarnykh and Yuan, 2004);

C. T2 mapping (T2-pSSFP method)

The pSSFP signal amplitude $S\phi$ acquired at a flip angle α and phase increment ϕ is a function of the longitudinal (T_1) and transverse (T_2) relaxation times and repetition time TR. An accurate T_2 estimation can be obtained from two pSSFP acquisitions as

$$T_2 = \frac{T_2^{estim}}{1 - 3/2 \cdot \eta \cdot T_2^{estim} / T_1}$$

where

$$T_2^{estim} = \frac{2TR}{\xi}\sqrt{\frac{S_{\phi 1}^2 - S_{\phi 2}^2}{S_{\phi 2}^2 \phi_2^2 - S_{\phi 1}^2 \phi_1^2}}$$

$\eta = 0.5\,(1 + \cos\alpha)/(1 - \cos\alpha)$. The term ξ must be determined numerically via a continued fraction expansion (Ganter, 2006).

Bibliography

Ganter C. Steady state of gradient echo sequences with radiofrequency phase cycling: analytical solution, contrast enhancement with partial spoiling. Magn Reson Med 2006;55(1):98-107.

Yarnykh VL. Fast macromolecular proton fraction mapping from a single off-resonance magnetization transfer measurement. Magn Reson Med. 2012;68: 166–178. doi:10.1002/mrm.23224

Yarnykh VL, Yuan C. Cross-relaxation imaging reveals detailed anatomy of white matter fiber tracts in the human brain. Neuroimage 2004;23:409–424.
